# Potential Interplay between Hyperosmolarity and Inflammation on Retinal Pigmented Epithelium in Pathogenesis of Diabetic Retinopathy

**DOI:** 10.3390/ijms19041056

**Published:** 2018-04-02

**Authors:** François Willermain, Lisa Scifo, Célia Weber, Laure Caspers, Jason Perret, Christine Delporte

**Affiliations:** 1 Department of Ophthalmology, CHU Saint-Pierre and Brugmann, 1000 Brussels, Belgium; fwillerm@ulb.ac.be (F.W.); lisa.scifo@ulb.ac.be (L.S.); celia.weber@ulb.ac.be (C.W.); lcaspers@ulb.ac.be (L.C.); 2 Institut de Recherche Interdisciplinaire en Biologie Humaine et Moléculaire (IRIBHM), Université Libre de Bruxelles, 1050 Brussels, Belgium; 3 Laboratory of Pathophysiological and Nutritional Biochemistry, Université Libre de Bruxelles, 1050 Brussels, Belgium; jason.perret@ulb.ac.be

**Keywords:** diabetic retinopathy, high salt diet, hyperosmolarity, retinal pigmented epithelium, blood retinal barrier, inflammation

## Abstract

Diabetic retinopathy is a frequent eyesight threatening complication of type 1 and type 2 diabetes. Under physiological conditions, the inner and the outer blood-retinal barriers protect the retina by regulating ion, protein, and water flux into and out of the retina. During diabetic retinopathy, many factors, including inflammation, contribute to the rupture of the inner and/or the outer blood-retinal barrier. This rupture leads the development of macular edema, a foremost cause of sight loss among diabetic patients. Under these conditions, it has been speculated that retinal pigmented epithelial cells, that constitute the outer blood-retinal barrier, may be subjected to hyperosmolar stress resulting from different mechanisms. Herein, we review the possible origins and consequences of hyperosmolar stress on retinal pigmented epithelial cells during diabetic retinopathy, with a special focus on the intimate interplay between inflammation and hyperosmolar stress, as well as the current and forthcoming new pharmacotherapies for the treatment of such condition.

## 1. Introduction

Anatomically, from front to back, the eye is composed of the cornea, the iris, the crystalline lens, the vitreous body, and the retina. The retina is made-up of ten histological layers that are composed of particular cell types (Table 1) [1]. The outer layer of the retina, the retinal pigmented epithelium (RPE), is separated from the choriocapillaris by the Bruch’s membrane. The functional role of the retina is to convert light energy into electrical impulses that are subsequently transmitted by the optic nerve to the brain, capable of interpreting and control our sense of vision.

Under physiological conditions, a blood-retinal barrier (BRB), structurally made of the inner BRB (iBRB) and outer BRB (oBRB) protects the retina by regulating ion, protein, and water flux into and out of the retina. In particular, RPE transcellular water flux, through aquaporin water channels, flows from the retina to the choriocapillaris in response to the presence of a transepithelial osmotic gradient. The iBRB refers to the barrier made by tight junctions between retinal capillary endothelial cells covered by astrocytes, pericytes, and Müller cells endfeet. The oBRB refers to the barrier resulting from the tight junctions between RPE cells [2]. 

Either iBRB or oBRB rupture can occur during the course of several ocular pathologies, including diabetic retinopathy (DR). BRB rupture results in increased osmotic pressure in the retina, leading to important water accumulation and macular edema formation that can impair sight. Under these conditions, we have hypothesized that RPE cells are likely to be subjected to hyperosmolar stress (HOS) at their apical membrane [3]. In addition, RPE cells might also be subjected to hyperosmolar stimulus through an increase of plasma osmolarity. In this context, it is relevant to note that high glucose-induced hyperosmolarity seems to promote angiogenesis and retinopathy through activation of the transcription factor tonicity-responsive binding-protein (TonEBP)/nuclear factor of activated T-cells 5 (NFAT5) [4]. In addition, recent studies suggest that age-related increase in plasma osmolarity could aggravate age-related macular degeneration (AMD), by promoting inflammation and angiogenesis within the retina [5]. Indeed, increased extracellular osmolarity could elicit the secretion of proinflammatory cytokines and angiogenic factors in RPE cells by activating different intracellular signaling pathways like MAPK and transcription factors such as NAFT5 [5]. Similarly, in diabetic rats, high salt diet (HSD) promotes intracellular edema in ganglion cells via an increased expression of aquaporin-4, and could therefore play a role in retinal edema formation [6]. However, in human, a study conducted on a large cohort of patients with type 2 diabetes, showed no association between HSD, an important contributor to plasma osmolarity, and an increased risk of DR, whereas the study showed an association between HSD and elevated incidence of cardiovascular disease [7]. 

On the other hand, HSD has also been associated with direct deleterious effects on the cardiovascular system dependently or independently of high blood pressure [8,9]. Association between AMD and cardiovascular diseases and hypertension have been reported [5,10]. In addition, hypertension contributes to an increased risk of developing glaucoma, and represents the major secondary risk factor of DR [11,12]. Furthermore, HSD has been shown to drive autoimmune disease and to exacerbate experimental autoimmune disease by inducing the activation of IL-17-producing CD4^+^ helper T cells (Th17) [13,14,15,16,17]. Indeed, Th17-IL-17 axis has been shown to play a role in the pathogenesis of diabetes and diabetic retinopathy (DR), as well as in autoimmune uveitis [18,19,20,21]. Therefore, it is likely that HSD and HOS could play a role in several ocular pathologies.

The present review aims at summarizing the current knowledge concerning the effects of HOS on RPE cells and possible role in the pathogenesis of DR

## 2. What Are the General Characteristics and Functions of RPE Cells?

The assembly of RPE cells, adhering by tight junction, form an RPE exhibiting 10-fold higher paracellular electrical resistance than the transcellular electrical resistance, a property defining the RPE as the oBRB [22]. The RPE is characterized by an apical membrane possessing long microvilli facing the light-sensitive outer segments of the photoreceptors cells, and a basolateral membrane facing the choriocapillaris [23,24]. RPE cells exchange nutrients, metabolic end products and signal molecules with photoreceptor cells, choroidal endothelial cells, and the blood stream [25].

**Figure 1:**
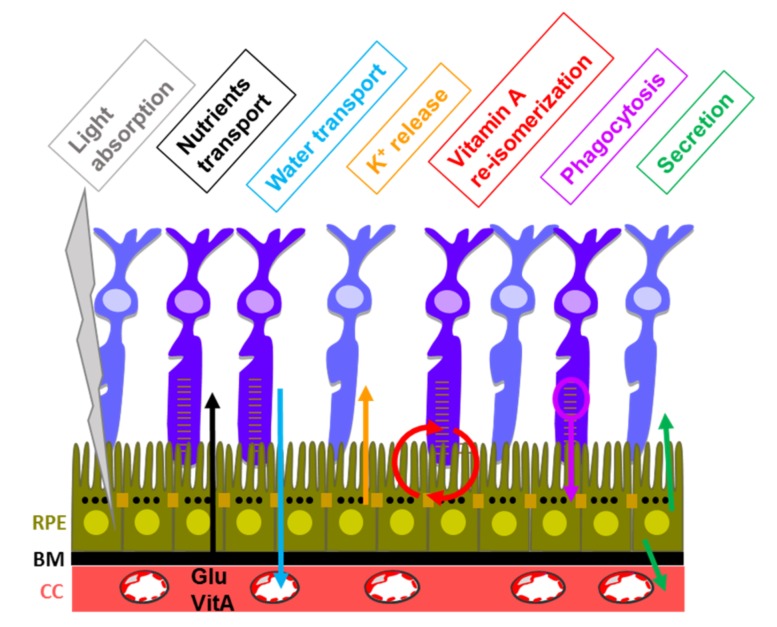
Functions fulfilled by the RPE in the process of vision. BM: basement membrane; CC: choriocapillaris; Glu: glucose; VitA: vitamin A; RPE: retinal pigmented epithelial cells. The arrows indicate nutrients transport (black arrow), water transport (blue arrow), K^+^ release (orange arrow), vitamin A re-isomerization (red arrow), phagocytosis (purple arrow) and secretion (green arrow).

The RPE fulfills important functions for the process of vision (Figure 1) including light absorption, nutrients transport (such as vitamin A and glucose) from blood to photoreceptor cells, transcellular water flux from the retina to the blood stream of the choriocapillaris, K^+^ release to the apical subretinal space to ensure constant electrical excitability of the photoreceptors, re-isomerization of all-trans retinal into 11-*cis trans* retinal, phagocytosis of altered photoreceptors, secretion of growth factors such as pigmented epithelium derived growth factor (PEDF) and vascular endothelial growth factor (VEGF) and immunosuppressive factors such as TGF-β maintaining the immune privilege of the eye and interfering with signaling pathways controlling the immune system [26,27,28].

In conclusion, RPE forms the oBRB, exchanges information with neighboring cells and fulfills crucial roles to ensure proper vision. 

## 3. How Is Water Present in the Subretinal Space, Eliminated by the RPE Cells?

Under physiological condition, water accumulates in the subretinal space due to the metabolic activity of the photoreceptors (for instance, the oxidative degradation of glucose produces water) and the intraocular pressure driving water movement from the vitreous body [29]. The RPE ensures transcellular water flux from the subretinal space to the blood stream of the choriocapillaris [26,29]. The transcellular water flux across the RPE is mainly driven by a transepithelial transport of Cl^−^ from the subretinal space to the blood.

RPE is likely subjected to light-dependent osmotic challenge as photoreceptors alter the composition of the subretinal space by constantly modifying its K^+^ concentration [29]. However, the precise extent and timing of dynamic volume changes encountered by the RPE cells remain poorly understood. Upon light exposure, the conformational change of rhodopsin to meta-rhodopsin sequentially activates transducin and a phosphodiesterase that hydrolyzes cyclic GMP into 5′GMP. The latter event leads to an inhibition of non-specific cation channels and photoreceptor hyperpolarization concomitant to a decrease in photoreceptor K^+^ efflux [26,29]. The resulting decrease in subretinal extracellular K^+^ concentration induces, within the RPE cells, a subsequent inhibition of the Na^+^–K^+^–2Cl^−^ cotransporter located at the apical membrane, activation of the inward rectifier K^+^ channels, and K^+^ extrusion within the subretinal space. The latter event compensates for the decrease in subretinal extracellular K^+^ concentration induced upon exposure to light. In the dark, K^+^ exits the photoreceptors. In addition, the generation of both Na^+^ and K^+^ gradient from the extracellular to intracellular compartment activate the apically-located Na^+^–K^+^–2Cl^−^ cotransporter in the RPE cells. The resulting increase in intracellular RPE Cl^−^ concentration provides the driving force for the Cl^−^ extrusion at the basolateral membrane by different anion or chloride channels [29]. Cl^−^ absorption through RPE is accompanied by the transport of water to balance osmotic pressure.

Transepithelial water flux across the RPE is ensured by the presence of transmembrane channel proteins permeable to water, namely aquaporins (AQPs) that allow water to move in response to osmotic gradients [30]. So far, thirteen mammalian AQPs have been cloned and classified as: (a) classical AQPs, only permeable to water (AQP0, AQP1, AQP2, AQP4, AQP5, AQP6, AQP8); (b) aquaglyceroporins, permeable to small solute such as glycerol and urea in addition to water (AQP3, AQP7, AQP9, AQP10); (c) unorthodox AQPs (AQP11, AQP12) [30]. AQPs expression in RPE cells is variable according to species. Table 2 summarizes the AQPs expression in rat and human RPE cells (Table 2). In addition, AQP1, AQP3, AQP4, AQP5, AQP6, AQP7, AQP10, AQP11, and AQP12 have been shown to be expressed in RPE cells derived from human embryonic (hESC) and human induced pluripotent stem cells (hiPSC) [31]. 

As a consequence of photoreceptor metabolic activity and the intraocular pressure, water accumulates in the subretinal space. RPE ensures water movement, through AQPs, from the subretinal space to the choroid to maintain retinal adhesion necessary for proper vision.

## 4. What Is the Role of Inflammation in BRB Rupture Occurring during DR?

In the USA, DR may arise in 86% of patients suffering from type 1 diabetes and 40% of patients suffering from type 2 diabetes [44,45]. The major consequences of DR are microvascular alterations and rupture of iBRB and/or oBRB that lead clinically to the formation of macular edema [46,47]. The mechanisms underlying this sight threatening disease have been the subject of intense investigations. A wide range of pathways, with evident cross talks, have been proposed to support DR at a molecular level: polyol pathway, advanced glycated-end products, protein kinase C pathway, oxidative stress, the renin-angiotensin system, epigenetics, or VEGF for example [45,48]. Recently, particular attention was paid to the role of inflammation in DR. 

The polyol pathway, a secondary pathway for glucose metabolism, is activated upon persistent elevated extracellular glucose levels [49]. This pathway, reduces glucose to sorbitol by aldose reductase, and sorbitol is eventually metabolized to fructose by sorbitol dehydrogenase [49]. Intracellular sorbitol accumulation induces osmotic damage of the retinal vascular cells and RPE cells, loss of pericytes, increased basement membrane thickness, and oxidative stress [49]. Increased polyol pathway activity may contribute to iBRB rupture during DR [49]. Advanced glycated-end products represent proteins and lipids that have been post-translationally modified by non-enzymatic glycation and oxidation following exposure to aldose sugars, such as glucose [50]. Advanced glycated-end products have been involved in the development of DR, as they modify hormones, cytokines, and extracellular matrix and lead to retinal vascular damage [51,52]. Hyperglycaemia-induced elevated diacylglycerol concentration activate the protein kinase C pathway, affecting multiple cellular processes that ultimately lead to increased retinal vascular permeability and abnormal angiogenesis [51]. Oxidative stress, resulting from hyperglycaemia induces production of reactive oxygen species, that can activate other molecular mechanisms involved in DR [47,53] The renin-angiotensin system has been suggested as playing a role in DR by damaging both neuronal and retinal vascular cells [54]. Diabetes-induced dysregulation of epigenetic modifications, such as DNA methylation, histone acetylation, and post-transcriptional RNA regulation, is involved in impaired expression of several genes (e.g., some genes involved in oxidation, angiogenesis, extracellular matrix degradation, etc.) and subsequent alteration of retinal vascular cells function in DR [55,56]. Several inflammatory factors, growth factors, and angiogenic factors have been shown to be involved in DR [57,58,59,60]. In animal models of DR and in humans suffering from DR, numerous stigmata of inflammation have been described: e.g., leukostasis, neutrophil and macrophage infiltration, complement and microglial activation, upregulation of cytokines, increased blood flow, vascular permeability, and tissue edema [52,57,58,59]. Intravitreal steroid administration have been useful to reduce macular edema and BRB rupture ensuing during DR and emphasizes the role of inflammation during DR development [61,62]. 

VEGF, and especially VEGF-A, can increase retinal ICAM-1 expression, vascular permeability, leukostasis, and BRB breakdown [63]. Anti-VEGF drugs have emerged as a useful pharmacotherapy to treat DR, but have limitations in terms of repeated injections, treatment burdens, and complete efficacy [64]. Hyperglycemia-induced VEGF expression increase in rat retina is mediated by protein kinase Cβ/human antigen R [65] and phospholipase A2 [66] pathways. Lipid-based nanocarriers containing siRNA silencing HuR expression [67] and phospholipase A2 inhibitors [66] could represent additional pharmacological tools to manage DR as they block the increase in retinal VEGF levels found in diabetic rats.

In this context of inflammation, hyperglycemia affects Tumor necrosis alpha (TNFα) and interleukin 6 (IL6) plasma levels [68], as well as local VEGF levels [69], while retinal hypoxia induce the release of cytokines, chemokines, and growth factors from macrophages and microglia [70]. Hypoxia also induces the expression of angiogenic factors, including VEGF and erythropoietin [70,71]. In addition to inflammatory cells and other cell types, RPE cells have been shown to express and secrete multiple cytokines, chemokines, and angiogenic factors, amongst others: IL6, IL8, MCP-1, TGFβ, and VEGF [72]. Alteration of RPE secretome, and in particular the release of inflammatory cytokines, occurring during DR seems to play a role in disease manifestations and progression [72]. 

Monocyte chemoattractant potein-1 (MCP-1, also known as chemokine ligand 2 (CCL2)), induces monocyte and macrophage infiltration of the retina and VEGF expression [73,74]. The role of MCP-1 in BRB alteration has been confirmed in MCP-1 knockout mice rendered diabetic. Indeed, these mice exhibited reduced retinal vascular leakage, monocyte infiltration, and microglial activation in the retina, as compared to wild-type mice also made diabetic [75]. 

TNFα, a cytokine transcriptionally regulated by NF-κB and secreted by macrophages and T cells, has been shown to stimulate leukostasis, as well as late BRB breakdown during DR [76]. TNFα induces the expression of adhesion molecules, leukocyte recruitment, apoptosis, chemoattraction of monocytes, growth factors (including VEGF) and other inflammatory mediators [63]. Systemic delivery of anti-TNFα has been shown to reduce the loss of pericytes and capillary degeneration in diabetic mice (Behl et al., 2008), and also leads to visual improvement of diabetic macular edema in humans [77].

Other inflammatory cytokines have been shown to play a role in DR. IL6 has been shown to induce vascular permeability and angiogenesis in DR [78,79] and stimulate VEGF expression [80]. IL8 probably plays chemoattractive and angiogenic roles in DR [59]. IL1β, mainly produced by macrophages, can activate NF-κB transcription factor involved in the transcriptional control of inflammatory cytokines, such as IL6 and IL8, in RPE cells [81]. IL1β also promotes angiogenesis and neovascularization [82]. Il1β receptor deletion in diabetes-induced mice protected mice from DR development as compared to wild-type diabetes-induced mice [83]. 

Toll-like receptors (TLRs), a conserved family of receptors responding to various microbes and endogenous ligands, are expressed in multiple retinal cells, including glial cells, RPE cells, photoreceptor cells, and endothelial cells [84]. Activation of TLRs are involved in DR by initiating intracellular signaling cascades leading to the production of proinflammatory cytokines, regulation of co-stimulatory molecules, oxidative damage of DNA, and secretion of angiogenic growth factors [84,85]. HMGB1 is a protein stabilizing the formation of nucleosome and gene transcription that can also bind to TLR2, TLR4, and receptor for advanced glycated-end products [84]. HMGB1 can be secreted by monocytes, activated macrophages, natural killer cells, mature dendritic cells, and endothelial cells, and acts as a proinflammatory cytokine [86]. In addition, HMGB1 is expressed by a variety of cells types within the retina: ganglion cells, photoreceptors, and RPE cells [87,88]. Secreted HMGB1 induces the expression of VEGF, TNFα, MCP1, and ICAM-1 and thereby accelerates the vasculopathy occurring in DR [89]. HMGB1 has been suggested to participate to the pathogenesis of DR due to its high levels in patients with proliferative diabetic retinopathy [87]. Furthermore, HMGB1 induced in vitro cytotoxic effects on glial cells, which contributed to pericytes and endothelial cells death [90]. High glucose levels induced the production of HMGB1 in RPE cells, which in turn activated NF-κB and VEGF production [91]. In retinal endothelial cells, high glucose levels also increased TLR2 and TLR4 expression, activated NF-κB, and increased levels of IL8, TNFα, MCP1, and adhesion molecules [92]. In type 2 diabetic rat retina, the expression of receptor for advanced glycated-end products, TLR2, TLR4, and HMGB1 were increased [91]. In DR, it could be inferred that HMGB1 might regulate VEGF production via TLR4 in RPE cells [91] and cytokine production via TLR2 and TLR4 in endothelial cells [92]. Using TLR7 knockdown mice, it has been shown that TLR7 could be involved in DR via an inflammatory response [93]. A recent study showed that purinergic P2X7 receptors are involved in high glucose-induced IL1β production in human retinal pericytes [94]. From this study, the authors inferred that in vitro exposure of human retinal pericytes to high glucose led to cell lysis and ATP release, with subsequent activation of P2X7 receptors and inflammasome (a caspase-activating multimeric complex processing/activating IL1β) [94]. Consequently, P2X7 receptors may represent a novel pharmacological target for managing the early phase of DR [94]. 

The roles of additional cytokines, chemokines, and growth factors in the pathogenesis of DR have been reviewed elsewhere [59].

The obvious interplay between distinct molecular mechanisms involved in the pathogenesis of DR renders understanding very complex. In this respect, while inflammation is undoubtedly now well recognized as an important player in the pathogenesis of DR, many lines of evidences suggest an intimate link between inflammation and angiogenesis, as well as inflammation and other pathways involved in DR. However, molecular mechanisms underlying the inflammatory pathways have not yet been fully deciphered. Nevertheless, the advances made thus far in our understanding of DR pathogenesis have led to the use of new molecules in the treatment of DR, such as anti-VEGF agents. Therefore, novel pharmacotherapies for the treatment of DR are likely to emerge from a deeper knowledge of the molecular mechanisms underlying the inflammatory pathways.

## 5. What Are the Effects of HOS on Innate and Adaptive Immune Responses?

HOS has been shown to alter both innate and adaptive immune responses. 

In mononuclear cells, HOS induced the expression and release of proinflammatory cytokines [95,96], chemotaxis [97], and inflammasome activation leading to IL1β secretion [98,99]. HOS also reduces the activation of IL4- and IL13-activated macrophages [100]. In human subjects, high salt intake induced an increase in monocytes number and proinflammatory cytokines IL6 and IL23 levels, as well as a decrease in anti-inflammatory cytokine IL10 levels [101]. 

Increased local salt concentrations boosted the induction of murine and human CD4^+^ T cells differentiation into Th17 phenotype [14,102] and thereby drives autoimmune diseases [14,15,103,104,105,106]. HOS also inhibits the suppressive function of FoxP3^+^ Tregs by increasing IFNγ secretion and inducing polarization towards Th1 phenotype [107]. 

Thereby, HOS has been shown to module both innate and adaptative immune responses.

## 6. How Can RPE Cells Be Subjected to HOS during DR?

**Figure 2:**
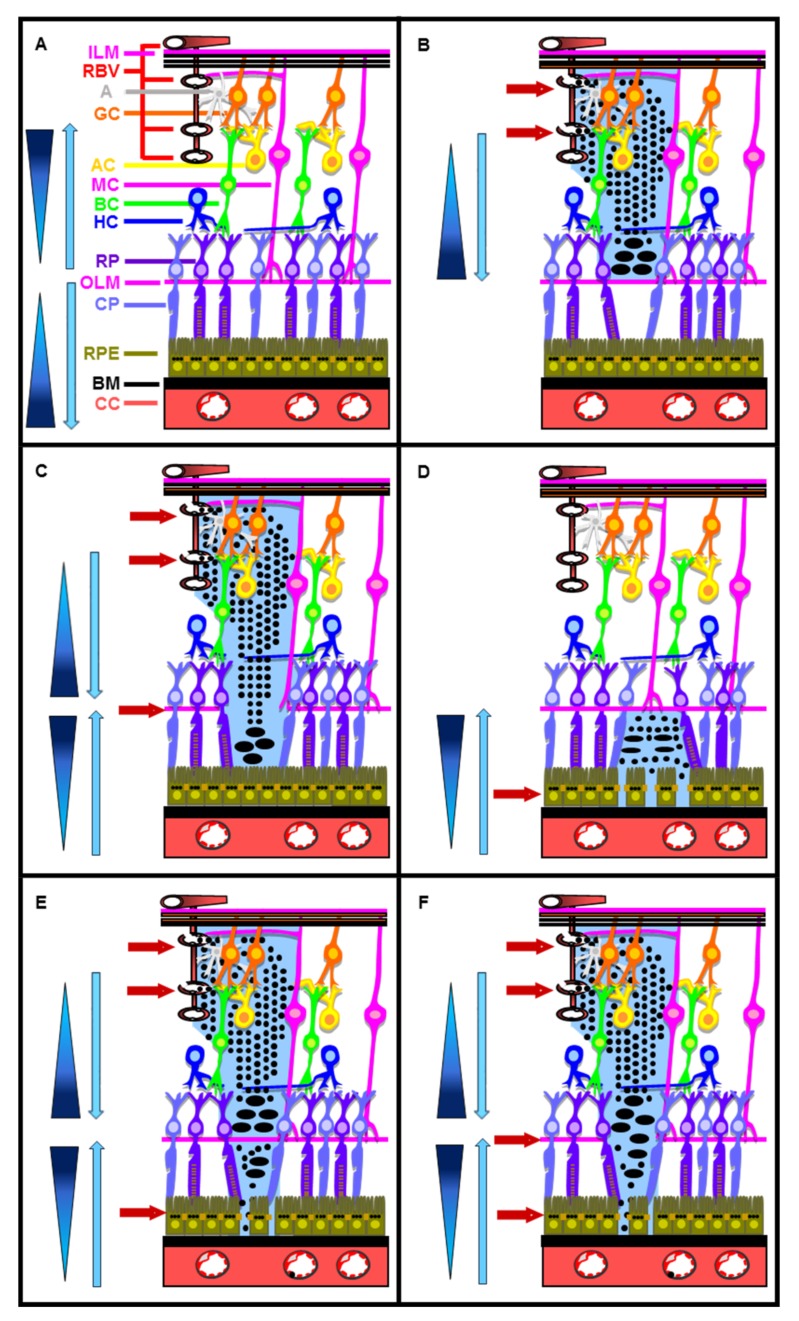
Intact and all theoretical blood retinal barrier BRB ruptures during diabetic retinopathy (DR). Schematic representation of: (**A**) intact iBRB and oBRB; (**B**) iBRB rupture without concomitant OLM rupture; (**C**) iBRB rupture with concomitant OLM rupture; (**D**) oBRB rupture; (**E**) iBRB rupture and oBRB rupture without concomitant OLM rupture; (**F**) iBRB and oBRB rupture with concomitant OLM rupture. Red horizontal arrows indicate the site of barrier rupture. Blue triangles indicate the osmotic gradient. Blue vertical arrows indicate the direction of water flux. A: astrocytes; AC: amacrine cells; BC: bipolar cells; BM: basement membrane; CC: choriocapillaris; CP: cone photoreceptors; GC: ganglion cells; HC: horizontal cells; ILM: inner limiting membrane; MC: Müller cells; OLM: outer limiting membrane; RBV: retinal blood vessel cells; RP: rod photoreceptors; RPE: retinal pigmented epithelial cells.

Under physiological conditions, the retina is protected by the iBRB and oBRB (Figure 2A). All theoretical BRB ruptures that can occur during DR are schematically represented in panels B to F in Figure 2. 

DR can frequently induce the rupture of the iBRB without (Figure 2B) or with (Figure 2C) concomitant outer limiting membrane (OLM) rupture. As a result of iBRB rupture, proteins, ions, and fluid diffuse within the subretinal space, from the damaged retinal vessels toward the outer retina. Proteins accumulate at the proximity of either the OLM, in case of iBRB rupture without concomitant OLM rupture (Figure 2B), or the RPE, in case of iBRB rupture with concomitant OLM rupture (Figure 2C). The respective consequences of such alterations are neuroretinal and subretinal edema, or neuroretinal edema alone. The accumulation of proteins induces protein precipitation and additional protein accumulation, as well as the persistence of an edema. 

DR can also induce the rupture of the oBRB alone [108,109] (Figure 2D). Upon such an oBRB rupture, proteins, ions, and fluid leak from the lumen of the choriocapillaris into the subretinal space, between the OLM and the RPE. The accumulation of proteins induces protein precipitation and additional protein accumulation, as well as the persistence of an edema.

In addition, iBRB rupture without concomitant OLM rupture combined with oBRB rupture (Figure 2E), or iBRB rupture with concomitant OLM rupture combined with oBRB rupture (Figure 2F) can also, at least theoretically, occur during DR.

Therefore, we hypothesized that RPE cells can be subjected to HOS during DR following either iBRB rupture with concomitant OLM rupture (Figure 2C), oBRB rupture alone (Figure 2D), iBRB and oBRB rupture without concomitant OLM rupture (Figure 2E), or iBRB and oBRB rupture with concomitant OLM rupture (Figure 2F) [3]. 

In addition, as mentioned earlier, it has been suggested that RPE cells can be subjected to HOS through increased local extracellular osmolarity following intake of dietary salt, an important cause of systemic hypertension, a major comorbidity factor of DR [110,111].

In conclusion, under a host of various conditions, RPE can be subjected to HOS during DR.

## 7. What Are the Consequences of HOS on the RPE?

The general mechanisms of cell response to HOS have been deciphered following extensive studies using renal medullary cells, that are physiologically exposed to HOS [112]. Cells exposed to HOS encounter a rapid cell shrinkage followed by the activation of regulatory volume increase (RVI) and subsequent volume recovery by swelling [113]. RVI can be divided into an early phase involving the activation of the Na^+^–K^+^–2Cl^−^ cotransporter and Na^+^/H^+^ exchangers, and a late phase involving the activation of the transcription factor TonEBP/NFAT5 that transactivates osmoprotective genes [112,113]. The activation of osmoprotective genes, such as aldose reductase, taurine transporter, sodium myo-inositol cotransporter, betaine/gamma-aminobutyric acid transporter, lead to the intracellular accumulation of organic osmolytes that contribute to the RVI [112]. In response to HOS, cells can undergo additional effects including cell cycle arrest, DNA damage, apoptosis, mitochondrial depolarization, alteration of transcription and translation machineries, oxidative stress, cytoskeleton rearrangement, and modulation of stress proteins [112]. In addition, HOS has been shown to play a role in inflammation by inducing cytokine production following the activation and transactivation activity of TonEBP/NFAT5 [114]. As RPE cells are likely to be subjected to HOS during DR, understanding the RPE response to HOS has recently become a subject of interest with respect to DR, as well as other ocular pathologies [3].

As mentioned above, RPE cells subjected to HOS have been shown to undergo an osmoadaptative response. Indeed, initially, HOS-induced shrinkage of RPE triggers an RVI response [115,116]. The HOS-induced increase in AQP3 [40] and AQP5 [42] expression and decrease in AQP4 expression [3] in RPE cells are probably involved in transcellular water flux occurring during RVI. The contribution of each AQP to the RVI remains to be addressed by performing single or combined AQP silencing. Secondly, HOS induces the activation of osmoprotective genes such as aldose reductase, an enzyme catalyzing the transformation of glucose into sorbitol, an organic osmolytes participating to RVI [117,118,119,120,121]. As aldose reductase is probably involved in adverse cellular effects of DR, HOS-induced increase in aldose reductase expression could thereby enhance DR. Clinical trials investigating the beneficial effects of aldose reductase inhibitors in the treatment of diabetic complications have shown little or no success [122,123,124]. Taurine transporter is also involved in the osmoregulation of RPE cells subjected to HOS [125]. HOS-induced increase in aldose reductase, taurine transporter and AQP5 mRNA levels has been shown to be dependent on TonEBP/NFAT5 transactivation activity, suggesting that targeting TonEBP/NFAT5 could represent a new therapeutic strategy for the treatment of DR [120,126,127]. 

HOS applied to the RPE cell layer can decrease its transepithelial electrical resistance (TER) [128], increase the ocular standing potential (positive wave) [129], and modify membrane and transepithelial potentials, and the amplitude of light-induced c-wave in electroretinograms [130,131]. 

Gene expression profiling in RPE cells subjected to HOS revealed a subset of genes that are typically involved in the regulation of cell proliferation [132]. HOS alters RPE cell number, proliferation and cell cycle phases, without affecting cell apoptosis and necrosis [132]. In agreement with the decreased percentage of cell number in G0/G1 and S phases and the increased percentage of cells in G2/M phase, decrease in cyclin D1 and B1 expression and activation of p38-mitogen-activated protein kinase have been shown in RPE subjected to HOS [132]. 

In RPE cells, HOS induced an increase in lysyl oxidase expression, an enzyme controlling the maturation of both collagen and elastin in the extracellular matrix [133]. Therefore, the increase in lysyl oxidase expression upon HOS might participate in the pathogenesis of proliferative retinopathy which may occur during DR. 

HOS can modify the RPE secretome by increasing the expression of VEGF [42], placental growth factor (PlGF) [127], monocyte chemoattractant protein-1 (MCP-1) [96], basic fibroblast growth factor (bFGF), and heparin-binding epidermal growth factor-like growth factor (HB-EGF) [134], interleukin 1β (IL1β), interleukin 18 (IL18), and moderately of epidermal growth factor (EGF), tumor-growth factor β1 (TGF-β1), interleukin-6 (IL6), and interleukin-8 (IL8) [134]. HOS has been shown to increase both IL1β and IL18 by inducing the priming of the Nod-like receptor protein 3 (NLRP3) inflammasome. Thus, during DR, as well as other ocular pathologies, in which BRB rupture and dietary habits lead to exposure of RPE cells to HOS, the latter seemingly prompts angiogenesis and inflammation by inducing the expression of angiogenic factors and proinflammatory mediators. 

The HOS-driven RPE responses include RVI, modification of electrical properties, alteration of cell cycle and proliferation, modulation of collagen and elastin maturation, and changes in RPE secretome prompting angiogenesis and inflammation.

## 8. Conclusions

Following BRB rupture that may occur during DR, or other ocular pathologies, RPE cells are probably subjected to HOS. HOS has been shown to induce a broad panel of responses in RPE cells. HOS can trigger an osmoadaptative response, modify the cell’s electrical properties, promote the maturation of collagen and elastin, reduce cell proliferation by inducing cell cycle arrest, and stimulate the secretion of angiogenic factors and proinflammatory mediators. The transcription factor TonEBP/NFAT5 appears to play a key role in host of HOS-mediated effects in RPE cells. Therefore, HOS likely contributes to the pathogenesis of DR and other ocular diseases characterized by a BRB rupture that can subject the RPE to HOS. Intimate interplay between inflammation and HOS is thus very likely involved in the pathogenesis of DR. Future new pharmacotherapies for the treatment of DR will arise from a deeper understanding of the molecular mechanisms underlying the pathogenesis of the disease.

## Figures and Tables

**Table 1:** Histological layers of the retina.

Layer’s No.	Layer’s Name	Layer’s Cell Types
1	Inner limiting membrane (ILM)	Müller cells (endfeet)
		Astrocytes
2	Nerve fiber layer (NFL)	Ganglion cells (axons)
		Retinal blood vessels cells
		Glial cells
3	Ganglion cell layer (GCL)	Ganglion cells (nucleus)
		Retinal blood vessels cells
		Glial cells
		Amacrine cells
4	Inner plexiform layer (IPL)	Bipolar cells
		Ganglion cells
		Amacrine cells
5	Inner nuclear layer (INL)	Bipolar cells (nucleus)
		Horizontal cells (nucleus)
		Amacrine cells (nucleus)
		Müller cells (nucleus)
6	Outer plexiform layer (OPL)	Photoreceptor cells
		Bipolar cells
		Horizontal cells
7	Outer nuclear layer (ONL)	Photoreceptor cells (nucleus)
8	Outer limiting membrane (OLM)	Photoreceptor cells
		Müller cells
9	Photoreceptor layer (PL)	Photoreceptor cells (rods and cones)
10	Retinal pigmented epithelium (RPE)	Retinal pigmented epithelial cells

Going from its inner to its outer part, the retina is made of ten histological layers numbered from 1 to 10. Each layer is composed of particular cell types.

**Table 2:** AQP expression in RPE cells.

AQP	Rat RPE	Human RPE
AQP0	[32,33]	-
AQP1	[32,33,34,35]	[36,37,38,39] controversy
AQP2	[34]	
AQP3	[33]	[40]
AQP4	[34,35,41]	-
AQP5	[32,33]	[42]
AQP6	[33,34]	-
AQP7	[33]	[43]
AQP8	[33]	[42]
AQP9	[32]	-
AQP10	-	-
AQP11	[32,33]	-
AQP12	-	-

Several aquaporins (AQPs) have been shown to be expressed in rat and human RPE cells: see references.

## Abbreviations

AQP	Aquaporin
AMD	Age-related macular degeneration
BRB	Blood retinal barrier
DR	Diabetic retinopathy
HOS	Hyperosmolar stress
HSD	High salt diet
iBRB	Inner blood retinal barrier
ILM	Inner limiting membrane
NFAT5	Nuclear factor of activated T-cells 5
TonEBP	Tonicity-responsive binding protein
oBRB	Outer blood retinal barrier
OLM	Outer limiting membrane
RPE	Retinal pigmented epithelium
